# Retraction: An Antifungal Mechanism of Protolichesterinic Acid from the Lichen *Usnea albopunctata* Lies in the Accumulation of Intracellular ROS and Mitochondria-Mediated Cell Death Due to Apoptosis in *Candida tropicalis*

**DOI:** 10.3389/fphar.2017.00882

**Published:** 2017-11-17

**Authors:** Frontiers Editorial Office

This paper is retracted by Frontiers. The publisher has discovered that the author(s) created and provided false information for the peer-review process. As the scientific integrity of the article cannot be guaranteed, and adhering to the recommendations of the Committee on Publication Ethics (COPE), the publisher therefore retracts the article.

The retraction of the article was approved by the Field Chief Editor of Frontiers in Pharmacology, Prof. Theophile Godfraind.

